# Topics of Public Interest

**Published:** 1918-12

**Authors:** 


### TOPICS OF PUBLIC INTEREST

**Army Casualties.** At about 50,000 total casualties in the American Expeditionary Force up to Oct. 18, the definite losses remain high in comparison with most other statistics of the kind: a trifle over a third dead, over 11% missing or known to be prisoners, while an allowance of less than 10% of non-returnable wounded brings the definite losses above the 50% line. The proportions hold good to date.

**German Birth Rate and Infant Mortality.** The live births for 1913 were 1,839,000; for 1914, 1,820,000; for 1915, 1,416,-000; for 1916, 1,103,000. The reduction of 40% is what might be expected under war conditions. These statistics are also of interest in connection with the rumor that Germany had deliberately underestimated her population in advance of the war. It will be noted that the 1913 (normal) birth rate is only a little over 25:1000 on a basis of the published population of about 67 million whereas our own birth rate is about 35:1000. Yet Germany has been regarded as a prolific nation and the United States, for a large part of its population, has been accused of race suicide. If as has been claimed by some, Germany had a population of 90 million, the 1913 birth rate would represent only a small fraction over 20:1000, which is about the minimum death rate of a country without marked immigration. Infant mortality had been reduced in Germany from 151:1000 in 1901 to 95:1000 in 1912. During the early stages of the war, it rose to 200 and, in places, to 300. Infantile marasmus is said to be common and rickets to occur in 23-43% of the infantile population. This is one of the details establishing the paradox that war does not change the proportion of the adult portion of the population to the degree that might be expected.

**Removal of Physicians to Cities.** Semi-official warning has been issued in regard to the exodus of physicians from small towns to cities, under the altered competitive conditions due to the war. As the entire medical profession is already being called upon for public service and as exemption, in case compulsory service becomes necessary will be based on the local needs of the population for medical attendance, it is obvious that steps may be taken to prevent removal. On the other hand, if the war should be ended soon, the discharge of medical reserve officers will remove the factor which tempts to such removals.

**Centenarian.** Mrs. Catharine Churchill, born in County Clare, Ireland, in 1814, was taken to the Chautauqua Co.

Hospital last year, on account of the enlistment of an adopted son. She jumped out of a window in an attempt to return to her home on a small farm and broke a leg. Almost complete restoration of function took place and she is now in good health. Mrs. Addison Burton of Ripley, aged 98, has recently cast her first vote, being probably the oldest woman voter in the state. She is closely followed by a Corning woman aged 95.

**Military Casualties and Deaths.** Up to Oct. 31, Australia had sent 336,000 troops from a population of 5,000,000, with a total of 290,191 casualties (nearly 90%) but only 54,431 deaths (a little less than 20%). The U. S. had sent to Europe about 2,200,000 troops from a population of over 100,000,000 (about 1,550,000 troops remaining in America), had sustained only 62,638 casualties (less than 3%) with 20,631 deaths (over 30%). The high percentage of our fatal casualties is difficult to explain.

**Birth Statistics.** The birth registration area, including 33 million, had a birth rate in 1916 of 24.8:1000 population, against a death rate of 14.7. The foreign born mothers comprised 66.3% in Connecticut, 52.84% in N. Y., down to 14.82 (whites only) in Md. The deaths under one year were 101:1000 in 1916, 100 in 1915. The range was from 70:1000 for Minn. to 121:1000 for Md. but only 101 for white infants. The mortality for children of Scandinavian mothers was 68, Polish 148, Negro 184. The mortality of male infants was decidedly higher than for females. The excess of births over deaths was about 1% for the whole area and up to about 2% for a few localities, while some showed a diminution on account of a large negro population.

**Volunteer Medical Service Corps.** The Council of National Defense has adopted the following classification: I. under 55, no physical disability, no dependents or adequate means. II. Same, not more than three dependents. III. More than three dependents or excluded from first two classes as needed by the community, an education institution, health dept. or some industry. (For example, by the commissioning of Dr. D. C. Main of Alfred, N. Y., Oct. 30, as Capt. M. C., this village is left without a physician). IV. Over 55 or disabled so as to be ineligible for commission. These will be recorded for possible future needs but will not be enrolled. State and County subdivisions of the work of the Council have been arranged, to facilitate enrollment.

Members of this corps are paid $200 a month traveling expenses and maintenance while on duty, for the present the payment being assumed by the American Red Cross. On Sept. 27, a call by Surgeon General Rupert Blue of the P. H. S. for 500 physicians to assist in caring for the influenza epidemic, was met within 72 hours and a second request for the same number resulted in a total of 1,135 by Oct. 1, names of volunteers continuing to come in so as to provide an adequate reserve list.

---

**Field Clerks and Nurses, Prisoners of War,** will, by a recent ruling, receive pay during captivity, the same as officers and soldiers.

